# Anaesthetic Management of A Newborn with Galactosaemia for Congenital Heart Surgery

**Published:** 2009-04

**Authors:** Arindam Choudhury, Sambhunath Das, Usha Kiran

**Affiliations:** 1 Senior resident, Department of Cardiac Anaesthesiology, Cardiothoracic Centre, All India Institute of Medical Sciences, New Delhi 110029; 2 Assistant Professor, Department of Cardiac Anaesthesiology, Cardiothoracic Centre, All India Institute of Medical Sciences, New Delhi 110029; 3 Professor and Head, Department of Cardiac Anaesthesiology, Cardiothoracic Centre, All India Institute of Medical Sciences, New Delhi 110029

**Keywords:** Galactosaemia, Newborn, Transposition of Great arteries, Congenital heart surgery, Anaesthetic management

## Abstract

**Summary:**

Galactosaemia is an autosomal recessive metabolic disorder that occurs due to galactose-1-phosphate uridyl transferase enzyme deficiency that leads to hepatic, ophthalmic, neural and renal derangements. Association of cyanotic congenital heart disease with galactosaemia is a rare occurence and a matter of great concern for the anaesthesiologist during open heart surgery. In this case report, the perioperative management of a newborn with galactosaemia operated for correction of transposition of great arteries (TGA) is discussed.

## Introduction

Galactosaemia is a rare autosomal recessive disorder that occurs due to galactose-1-phosphate uridyl transferase (GALT) enzyme deficiency.^1^ The physiologic consequences of this deficiency leads to accumulation of galactose in nervous tissue, liver, lens and kidney causing severe mental retardation, liver damage and cataract formation.^2^ Patient with galactosaemia who has hepatic involvement, may deteriorate further by cardiac surgery under cardio-pulmonary bypass (CPB).

The anaesthetic management of patient suffering from galactosaemia undergoing corrective surgery for congenital heart disease under CPB is not found in literature. We report the preoperative detection and mangement of galactosaemia in a newborn who was operated for correction of TGA.

## Case Report

A 32 week preterm male child was born to a second gravida healthy mother. He was delivered through an emergency lower segment caesarean section. There were signs of fetal distress and the liquor amnii was also meconium stained. At birth, the neonate's body weight was 1.75 kg. He was cyanosed and an echocardiography revealed presence of atrial septal defect (ASD), ventricular septal defect (VSD) and dextro-transposition of great arteries (d-TGA). He was planned for arterial switch operation (ASO) cum ASD and VSD closure. The new born was admitted to the cardiac surgery ward on 26th day of extra uterine life for routine preoperative workup. During his preanaesthetic assesment, we noticed a yellowish discolouration of conjunctiva on day 27 of life. He had generalised hypotonicity with decreased spontaneous activity. He appeared pale and his heart rate was 150/ min, sinus rhythm, peripheral pulses were just palpable and extremities were cold. There was an obvious central cyanosis evident in his lips. On examination, his abdomen was soft and a 2 cm liver was palpable below right costal margin. Spleen was not palpable. Preliminary cardiovascular examination revealed nomal first heart sound, single second heart sound and an ejection systolic murmur was audible in the left parasternal area. Auscultation of chest suggested bilateral clear vesicular breath sounds. On examination of central nervous system, the baby was found to be lethargic with very minimal spontaneous activity. He had generalised hypotonia but there was no abnormal posturing.

His serum chemistry reports were reviewed and total serum bilirubin was found to be 20.9 mg/dl with unconjugated fraction of 15.4 mg/dl, aspartate aminotransferase (AST) – 78 IU/L, alanine aminotransferase (ALT) – 34 IU/L and serum alkaline phosphatase (ALP) – 810 IU/L. Both the mother and the baby shared the same blood group B-Rh positive. Coomb's test was also done which was negative. Thus, any fetal hemolytic reaction was also ruled out. Detection of reducing sugar in his urine and hyperbilirubinemia gave us a clue for galactosaemia although a confirmatory test was also simultaneously ordered. Patient was then referred to the neonatologist for a thorough evaluation of jaundice and the patient was transfered to neonatal intensive care unit (NICU). Patient recieved intensive phototherapy and the mother was advised not to nurse her child with breast milk, instead a lactose free milk powder was prescribed (zerolac, Raptakos Brett, India - 30 ml 2 hourly). Syrup phenobarbitone was also prescribed 12.5 mg orally once daily. The diagnostic panel suggested positive blood galactosaemia screening test, G-6-PD screen negative, reticulocyte count 2% and cryoglobin negative. GALT enzyme assay was 0.01 U/g Hb (Normal range 15.00 - 35.00). After about one month intensive conservative treatment for galactosaemia induced hyperbilirubinemia, the preoperative liver function tests were improved to total serum bilirubin - 1.6mg/dl, AST - 48 IU/L, ALT - 30 IU/ L and ALP – 121 IU/L. Platelet count was 2.39,000/cu.mm, prothrombin time 20 second and partial thromboplastin time 41 second. He was posted for ASO with ASD-VSD closure. Informed parental consent was taken with special emphasis on the possibility of a prolonged ICU stay.

On the morning of surgery the child received ceftazidime 20mg.kg^−1^ and no anaesthetic premedication was given. After attaching ECG monitor and SpO2 probe, sevoflurane was administered via a face mask in titrated concentration. A peripheral venous access and a right femoral arterial line was secured for invasive blood pressure monitoring. Base line blood gas analysis, blood glucose estimation and activated clotting time were noted. His trachea was intubated with a 3.5 mm uncuffed endotracheal (ET) tube and the intubation was facilitated by atracurium 0.5 mg.kg^−1^, fentanyl 10 mcg and midazolam 0.5 mg. A triple lumen central venous catheter was inserted under all aseptic precaution into the left femoral vein and central venous pressure (CVP) was monitored. Intraoperatively, his blood glucose was monitored every half an hour. The maintenance fluid used before CPB was 5% dextrose and was administered at 10 ml/hr. Anaesthesia was maintained with 1-2% sevofluorane in air-oxygen mixture, top ups of atracurium for muscle relaxation and titrated doses of fentanyl for adequate analgesia during the surgery. The CPB circuit was primed with 0.9% saline, mannitol and packed red cell and blood cardioplegia was used to arrest the heart. The total CPB time was 96 minutes and the aortic cross clamp time was 59 minutes and the intraoperative blood loss was about 20 ml. After successful arterial switch operation on cold cardiplegia induced arrested heart, the child was safely weaned off bypass with dopamine and dobutamine infusions of 5 mcg.kg^−1^.min^−1^ each. All the necessary prerequisites were fulfilled before weaning from bypass. Post bypass electrolyte and blood gases were within normal limit. Activated clotting time (ACT) was normalised with protamine sulphate. One unit each of fresh frozen plasma (FFP) and platelets concentrate (PC) was transfused. Patient was shifted to the intesive care unit and mechanically ventilated. He had transient junctional atrial tachycardia and he remained haemodynamically unstable for sometime but he recovered when digitalis was started. Injection epsilon amino caproic acid 100mg / kg was administered prior to initiation of CPB and the same dose was repeated during and after CPB. PC and FFP were also transfused in the postoperative period to minimise surgical site bleeding. The total chest tube drainage after 24 hrs was 20 ml. Lactose free enteral nutrition was started after an initial restriction of feeds for five hours. Patient was gradually warmed with a radiant warming device and his intake and output was monitored on an hourly basis. The trachea was extubated on second postoperative day. Patient remained haemodynamically stable and maintained oxygen saturation after extubation inside an oxygen hood. The postoperative liver function tests were - total serum bilirubin 1.6mg/dl, AST 48 IU/L, ALT 30 IU/L and ALP 121 IU/L. Platelet count was 3,50,000/cu.mm, prothrombin time 12 second and partial thromboplastin time 36 second. He has been shifted to the ward on 4^th^ postoperative day with an advice to continue lactose free diet until further advice. The rest of his hospital stay was uneventful and he was discharged home after 3 days.

## Discussion

Galactosaemia is an inborn error of metabolism that occurs due to deficiency of GALT, galactokinase and uridine diphosphogalactose 4-epimerase enzyme deficiency. Classic galactosaemia occurs due to GALT deficiency and is associated with jaundice, weight loss, cataract formation, mental retardation and cirrhosis due to excessive accumulation of galactose in the respective tissues in late infancy or early childhood.^2^ Patients with galactokinase deficiency have jaundice only and mental retardation is not a characteristic feature in these children.^1^ Neonatal screening for galactosaemia is not routine in the Indian subcontinent. Most of the patients present in the early weeks of life as in our case although this child was primarily diagnosed to have congenital heart disease. During preanaesthestic assessment for a corrective surgery ford-TGA, a thorough evaluation of the patient has helped us to screen this patient for further evaluation and optimisation of jaundice before arterial switch operation (Jatene operation), a major surgery under cardio-pulmonary bypass. In this surgery, the great vessels are transected above the valves and moved to their opposite ventricles. The origins of the left and right coronary arteries are dissected off as individual buttons that are rotated into place and reimplanted into the neoaorta. The new pulmonary artery is reconstructed where the coronary artery buttons were removed. When the procedure is complete, the left ventricle receives oxygenated blood from the lungs, which it then pumps to the body. The right ventricle pumps the systemic venous return to the lungs in the usual fashions.

Poor feeding, vomiting, diarrhoea, jaundice, lethargy-hypotonia, hepatomegaly, encephalopathy, bleeding and excessive bruising are commonest signs and symptoms of galactosaemia.^3^ Our patient also had similar findings along with unconjugated and combined hyperbilirubinemia and abnormal liver function test. To prevent further damage from accumulation of its metabolites, lactose was totally eliminated from the child's diet during the preoperative period.^4^

Challenges faced during the anaesthetic management are numerous. Newborns with galactosaemia have elevated clotting time, that make the children prone for excessive bleeding during surgery. Institution of CPB mandates administration of an anticoagulant (viz. heparin) potentially increasing the incidence of bleeding in this group of patients. Also, albuminuria may cause osmotic diuresis in this group of patients making urine volume an unreliable measure of intravascular volume in them.^5^ Particular attention is required in maintaining asepsis while inserting invasive vascular catheters as these patients are prone to develop E coli neonatal sepsis.^6^ Special attention is given in avoiding anaesthetics that are either hepatotoxic (like halothane) or are metabolised by liver (e.g., pancuronium, thiopental, opioid analgesics). Hypotension during surgery was avoided to prevent hepatic and renal damage in an already derranged hepatic function. Further, a perfusion pressure of 35-45 mmHg was strictly maintained throughout the period of CPB.

In conclusion, presence of jaundice and reducing sugars in the urine of neonates may be a cause for suspicion of galactosaemia. Prevention of hepatic and renal damage by selecting antibiotics, anaesthetics and cardiac drugs that are not or less dependent on hepatic and renal metabolism; is of prime importance. Avoidance of lactose containing foods like breast milk, milk products, biscuits, breads and drugs is essential. Haemodynamic stability throughout the perioperative period and a timely corrective cardiac surgery is the key to successful management of galactosaemia with associated congenital heart disease.
